# Pyrite as a catalyst for the emergence of multiphase primitive cells

**DOI:** 10.3389/fmicb.2025.1747422

**Published:** 2026-01-08

**Authors:** Niannian Ding, Tianhao Ren, Dehai Liang, Hailiang Dong

**Affiliations:** 1 Center for Geomicrobiology and Biogeochemistry Research, State Key Laboratory of Geomicrobiology and Environmental Changes, China University of Geosciences, Beijing, China; 2 Beijing National Laboratory for Molecular Sciences, Department of Polymer Science and Engineering and the Key Laboratory of Polymer Chemistry and Physics of the Ministry of Education, College of Chemistry and Molecular Engineering, Peking University, Beijing, China; 3 Frontiers Science Center for Deep-time Digital Earth, China University of Geosciences, Beijing, China

**Keywords:** mineral catalysis, mineral-protocell interactions, multiphase coacervates, primitive cells, pyrite

## Abstract

Coacervate-based protocells are minimal systems that mimic certain properties of natural cells and are used to investigate the emergence of life from nonliving chemical systems. However, constructing protocells with hierarchical structures and life-like functions remains a challenge. In this work, we develop a novel coacervate-based protocell (droplet) composed of single-stranded oligonucleotides (ss-oligo), quaternized dextran (Q-dextran), and 3,3′,5,5′-tetramethylbenzidine (TMB). In the presence of natural pyrite, the droplet encapsulates the pyrite, enhancing its peroxidase-like catalytic activity. This activity catalyzes oxidation of TMB to its oxidized form (TMBox), inducing a transition from single-phase to multiphase droplets. The resulting multiphase droplet consists of an internal TMBox/ss-oligo phase and a surrounding Q-dextran/ss-oligo phase, facilitating the sequestration and partitioning of ss-oligo into discrete regions. Notably, these droplets exhibit stability in their internal sub-compartments during fusion, showing their potential as dynamic and functional models in synthetic biology and biotechnology applications. Our study highlights that the catalytic activity of minerals may serve as a potential strategy for constructing hierarchically structured protocells that resemble the morphology and functions of living cells. This finding represents a significant step towards improving our mechanistic understanding of the transition from non-living matter to living systems under prebiotic conditions.

## 1 Introduction

Coacervate droplets, which are formed by liquid–liquid phase separation (Zheng et al., 2021) and share the same mechanism for the formation of membraneless organelles in biological systems (Brangwynne et al., 2015; Shin and Brangwynne, 2017), serve as an ideal platform for constructing artificial cells (Cook et al., 2023) and exploring the possible pathways of origins of life. Characterized by molecularly crowded and highly dynamic viscoelastic interiors (Alshareedah et al., 2024), coacervate droplets exhibit remarkable abilities especially to selectively concentrate bioactive molecules and to enhance enzyme reactivity (Abbas et al., 2021; Strulson et al., 2012). The features similar to those in living cells (Lin et al., 2023) have been achieved by varying the components, tuning the morphologies (Spoelstra et al., 2020), and placing the coacervate droplets under different conditions. For example, the droplets undergo dynamic fusion, fission, assembly, and dissolution in response to salt/proton concentration (Hong et al., 2022; Karoui et al., 2021), temperature (Hong et al., 2022), and physical stimuli such as light (Lafon and Martin, 2021; Agarwal et al., 2023), electric or magnetic stimuli (Capasso Palmiero et al., 2020; Jing et al., 2020).

Dynamic behavior is a fundamental feature of all living cells, which has been one of the aims when designing coacervates droplets as protocell models (Martin, 2019; Nair et al., 2023). Biochemical reactions including phosphorylation/dephosphorylation reactions (Aumiller and Keating, 2016), oxidation of glucose (Karoui et al., 2021), and polynucleotide synthesis (Deshpande et al., 2019) have been exploited to control the formation and dissolution of coacervate droplets *in vitro*. However, the protein-based enzymes governing these processes are believed to be absent on primitive Earth before the origin of life. It is proposed that the minerals possessed the enzyme-like activity (Huang et al., 2024) and served as the earliest catalysts (Aithal et al., 2023). Studies have shown that the activity of mineral nanoparticles can be enhanced when encapsulated by protocells (Koga et al., 2011; Smokers et al., 2024). However, the coacervate protocells whose dynamic behaviors are regulated by mineral-catalyzed chemical reactions are rarely studied.

Pyrite (FeS_2_) was formed on the Hadean Earth before the origin of life (Golden, 2019; Golden et al., 2019). It has been shown that pyrite is able to produce hydrogen peroxide (H_2_O_2_) under anoxic conditions (Gil-Lozano et al., 2017; Borda et al., 2001). In addition, pyrite can further decompose H_2_O_2_ to generate highly reactive hydroxyl radicals (·OH). The peroxidase-like (POD-like) catalytic activity (Cao et al., 2024; Meng et al., 2021) of pyrite may have contributed to oxidative processes on the early Earth. In this study, we incorporated pyrite particles in the coacervate droplet formed by single-stranded oligonucleotide (ss-oligo)/quaternized dextran (Q-dextran)/3,3′,5,5′-tetramethylbenzidine (TMB), and investigated the catalytic properties of natural pyrite microparticles and its effect on the dynamic behavior of the droplets. We observed that the pyrite microparticles were effectively encapsulated by the coacervate droplets which enhanced the POD-like activity. As the TMB molecules were oxidized to TMBox by pyrite particles, the droplet underwent a transition from single-phase to multiphase, wherein the different proportions of TMBox, ss-oligo, and Q-dextran form membraneless sub-compartments. Significantly, these sub-compartments facilitate heterogeneous distribution and stability (the lower mobility) of DNA. Our findings elucidate the mutual interactions between coacervates and minerals, suggesting potential implications for the evolutionary processes of protocells on the early Earth.

## 2 Materials and methods

### 2.1 Materials

Two ss-oligos were purchased from Beijing Tsingke Biotech Co., Ltd., including a 60-nt ss-oligo-L (TAATAGCTAATCATAGAATCTCTTTTAAATATAAAGTCATATCAAATTAAGAGAGAATTG) with and without FAM-labeling at the 5′ end, and a 21-nt ss-oligo (CTTACGCTGAGTACTTCGATT) with and without TAMRA-labeling at the 5′ end. The unlabeled ss-oligo samples were dissolved in deionized water to obtain a stock solution of 6 mg/mL, then mixed with 6.0 × 10^−3^ mg/mL Fam-labeled ss-oligo-L or TAMRA-labeled ss-oligo to track their distribution during coacervate droplet formation and separation. Q-dextran and TMB (≥99%) were purchased from Aladdin (Shanghai, China) and dissolved in deionized water. Hydrochloric acid (HCl, 36.0%–38.0%), hydrogen peroxide (H_2_O_2_, 30%), sodium hydroxide (NaOH), sodium acetate (NaOAc), acetic acid (AcOH), disodium hydrogen phosphate (Na_2_HPO_4_), glycine, and sodium citrate (C_6_H_5_Na_3_O_7_) were all purchased from Sinopharm Chemical Reagent Co., Ltd. All chemicals were used as received without further purification. Deionized water was used throughout the experiments.

### 2.2 Preparation of pyrite microparticles

Recent studies have demonstrated that FeS_2_ exhibits a peroxidase-like (POD-like) activity (Cao et al., 2024; Meng et al., 2021), prompting our investigation into how the POD-like activity of pyrite affects droplet dynamics. Bulk pyrite was obtained from Taobao, a popular e-commerce platform in China. Pyrite microparticles (about 13 μm) were obtained by grinding and passing through a 1,000-mesh sieve. Pyrite purity was checked using X-ray diffraction (XRD) performed on a Rigaku-D/MAX-PC 2500 instrument, equipped with Cu Kα radiation and a graphite monochromator, operating at 40 kV and 200 mA. Samples were scanned over a 2-theta range of 3–70 with a step size of 0.02 and a counting time of 0.12 s per step.

### 2.3 Optimizing conditions for the peroxidase-like activity of pyrite

The POD-like activity of pyrite was measured using substrate TMB in the presence of H_2_O_2_ under different reaction conditions. All experiments were performed at 23 °C. First, experiments were carried out in acetic acid–sodium acetate (AcOH–NaOAc) buffer (200 mM, pH 3.6), containing 1.0 mg/mL TMB, 0.10 mg/mL pyrite, and/or 0.50 mM H_2_O_2_. After the reaction had proceeded for 10 min, the absorbance of the colored oxidation products of TMB was monitored across 300–1,000 nm using a multi-mode microplate reader (BioTek Synergy™ H4, USA). Although TMBox exhibits stronger absorbance peaks at shorter wavelengths, the 652 nm peak was chosen for subsequent kinetic analyses because it provides the most stable and interference-free signal.

To evaluate the influence of different buffer compositions on pyrite activity, experiments were conducted in 160 mM/80 mM/40 mM AcOH–NaOAc, glycine–HCl, or citric acid–disodium hydrogen phosphate (CAPS) buffer at pH 3.6. After 10 min, the absorbance at 652 nm was measured, confirming the highest activity in 40 mM glycine–HCl. To determine the optimal reaction pH, the glycine–HCl buffer was adjusted using HCl/NaOH to pH 1.50, 2.33, 3.10, 3.60, 4.07, 4.70, 5.31, 6.31, 7.61, 8.53, and 10.1. The optimal pH for pyrite activity was found to be 4.07. Thus, all subsequent experiments were conducted under the conditions of 40 mM glycine–HCl at pH 4.07. To assess the effect of pyrite concentration, experiments were conducted using pyrite concentrations of 0.15 mg/mL, 0.10 mg/mL, 0.080 mg/mL, 0.040 mg/mL, and 0.020 mg/mL.

For kinetic parameter determination, all experiments were carried out in glycine–HCl buffer (40 mM, pH 4.07). The pyrite-only group contained 2.0 mg/mL TMB, 0.15 mg/mL pyrite and a concentration gradient of H_2_O_2_ from 0 to 5.0 mM (0, 0.078, 0.16, 0.31, 0.62, 1.2, 2.5, and 5.0 mM). The droplet-encapsulated pyrite group contained 3.0 mg/mL unlabeled ss-oligo (21-nt), 3.6 mg/mL Q-dextran, 0.15 mg/mL pyrite, and 2.0 mg/mL TMB. After incubating for 5.0 min, the same concentration gradient of H_2_O_2_ was added to initiate the reaction. The kinetic parameters were measured at 652 nm, 10 min after H_2_O_2_ addition. The Michaelis–Menten constants were obtained by fitting the data to the Michaelis–Menten equation using GraphPad Prism (10.1.1):


$$v=(V_{\max}\times [S])/(K_{M}+[S])$$ (1)

where *v* is the initial reaction velocity, [*S*] is the substrate concentration, *V*_max_ is the maximal reaction rate observed at the saturating substrate concentration, and *K*_M_ is the substrate concentration at which the reaction rate is half of *V*_max_. *v* is calculated using the following Equation 2:


v=ΔA/(Δt×ε×l) (2)

where Δ*A* is the change of absorbance value, Δ*t* is the reaction time interval for the change (s), *ε* is the molar absorption coefficient of the colorimetric substrate, which is typically 39,000 M^−1^ cm^−1^ at 652 nm for oxidized TMB, and *l* is the path length of light traveling in the cuvette (0.2 cm in our experiments). The kinetic constants *K*_M_ and *V*_max_ were calculated in GraphPad Prism 10.1.1 software through fitting the *v* versus [*S*] curve using the Michaelis–Menten equation (Equation 1).

The catalytic constant (*k*_cat_) is defined as the maximum number of substrate molecules converted to product per unit of time and is calculated by the following Equation 3:


$$K_{\mathrm{cat}}=V_{\max}/[E]$$ (3)

where [*E*] is the concentration of pyrite (M).

*k*_cat_/*K*_M_, which characterizes the catalytic ability of pyrite to the substrate (TMB), reflects the catalytic efficiency of pyrite.

### 2.4 Coacervate droplet preparation and phasing assays

Unlabeled ss-oligo (21-nt, 3.0 mg/mL), Q-dextran (3.6 mg/mL), pyrite (0.30 mg/mL), TMB (0.30 mg/mL), and 0.10 mM H_2_O_2_ were incubated in glycine–HCl buffer (40 mM, pH 4.07), in a total volume of 10 μL. In the fluorescence observation experiments, an additional 6.0 × 10^−3^ mg/mL of FAM-labeled ss-oligo-L or TAMRA-labeled ss-oligo was added to facilitate the observation of DNA distribution. After incubation for 5.0 min at 23 °C to allow for formation of condensates, samples were visualized by brightfield microscopy and fluorescent microscopy at 100× magnification using a Zeiss Axio Observer Z1 Inverted Fluorescence Microscope (Germany).

Oxidized TMB exhibits absorbance across a broad wavelength range, which interferes with fluorescence imaging. Consequently, droplets with higher TMBox concentrations appeared darker under fluorescence microscope. To mitigate the color interference of TMBox on FAM-labeled ss-oligo-L/TAMRA-labeled ss-oligo, GSH (glutathione) was used to reduce TMBox to TMB. The final working concentrations of pyrite microparticle, TMB and H_2_O_2_ were 0.10 mg/mL, 0.10 mg/mL and 0.50 mM, respectively. GSH (0.40 mM) was added either at 0 or 6.0 min after the reaction had begun, and the change of peak intensity (652 nm) was monitored. The group without GSH was supplemented with deionized water.

### 2.5 Fluorescent recovery after photobleaching (FRAP) assay

To investigate the mobility of DNA across and within droplets, FRAP experiments were conducted on both single-phase and multiphase droplets. The standard assay (10 μL) contained 3.0 mg/mL unlabeled ss-oligo, 6.0 × 10^−3^ mg/mL TAMRA-labeled ss-oligo, 3.6 mg/mL Q-dextran, 1.0 mM H_2_O_2_ in glycine–HCl buffer (40 mM, pH 4.07). After incubation for 5.0 min at 23 °C to allow condensate formation, samples were imaged under the Super Resolution Microscope N-SIM S (Nikon A1 N-SIM S, Japan) using a 100 × oil-immersion objective. Condensates with a clear red fluorescent signal were selected for photobleaching (“automatic focus” was activated, “pixel binning” was set at 2 × 2, and “exposure time” was set at 300 ms). The partial region was photobleached at 561 nm (Laser Power: 17.6), followed by a fluorescence recovery period of 50.4 s. The fluorescence intensity of the photobleached area was normalized relative to that of the unbleached area.

## 3 Results

### 3.1 Characterization of pyrite POD-like activity

**Figure 1:**
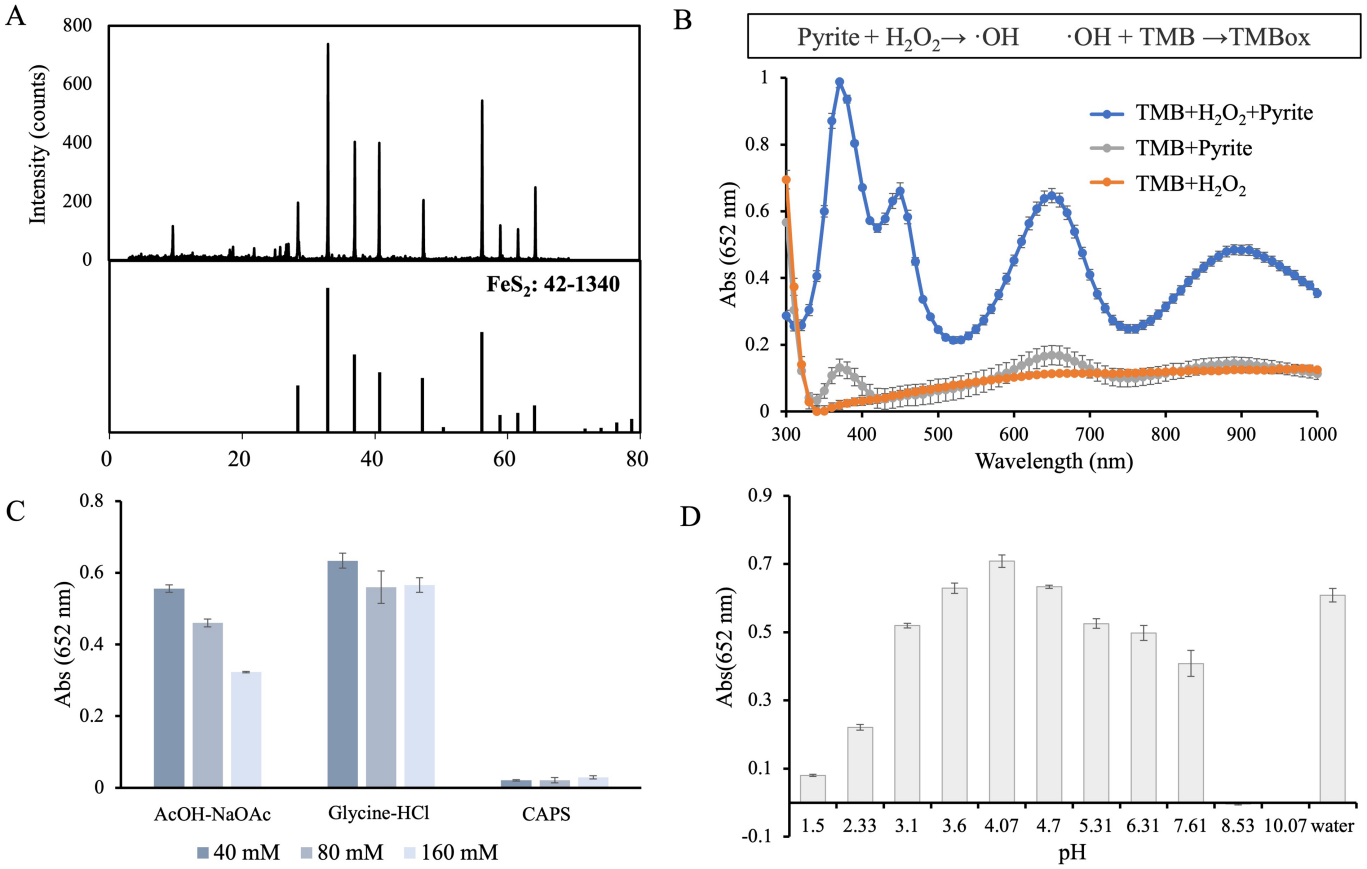
Characterization of pyrite. **(A)** XRD pattern of natural pyrite. **(B)** (Top) Reaction scheme of TMB oxidation catalyzed by pyrite. (Bottom) Formation of TMBox in the presence of pyrite (0.10 mg/mL) and/or H_2_O_2_ (0.50 mM). **(C)** Influence of buffer composition on the catalytic activity of pyrite (0.10 mg/mL). **(D)** Effect of pH on the catalytic activity of pyrite (0.10 mg/mL).

XRD patterns of pyrite matched the standard pyrite pattern [FeS_2_ (PDF 42-1340)], confirming its crystalline structure (Figure 1A). The POD-like catalytic activity was then investigated using TMB oxidation. In the sample containing pyrite, TMB and H_2_O_2_, a full scan absorption spectrum of TMBox displayed peaks at 380 nm, 450 nm, and 652 nm. No significant absorbance was detected when TMB was mixed with H_2_O_2_ without pyrite (Figure 1B). However, when pyrite was mixed with TMB without H_2_O_2_, a weak absorbance peak corresponding to TMBox was observed (Figure 1B). This is consistent with previous reports (Cohn et al., 2006; Gil-Lozano et al., 2017; He et al., 2023), indicating that when pyrite is exposed to water, a small amount of H_2_O_2_ is spontaneously generated. The resulting hydroxyl radicals subsequently react with TMB, forming trace amount of TMBox. Although the absorption peak at 652 nm is not the highest in intensity, it provides the most stable and interference-free signal for kinetic quantification. Therefore, 652 nm was selected as the detection wavelength.

To evaluate the POD-like activity of pyrite microparticles, we first determined the optimal buffer system for the reaction. Pyrite exhibited POD-like activity in both acetic acid–sodium acetate (AcOH–NaOAc) and glycine–HCl buffer systems, while no activity was detected in citric acid-disodium hydrogen phosphate (CAPS) buffer (Figure 1C), possibly due to adsorption of citric acid onto pyrite surface (Zhang et al., 2017). Furthermore, the catalytic activity was influenced by buffer concentration, with a relatively high activity around 40 mM. Considering that different buffer components may introduce additional effects, the catalytic activity of pyrite was subsequently evaluated in 40 mM glycine buffer, with the pH adjusted between 1.50 and 10.07 using HCl or NaOH, revealing the highest activity at pH 4.07 (Figure 1D).

### 3.2 The kinetics of pyrite-catalyzed oxidation: encapsulated and non-encapsulated states

**Figure 2:**
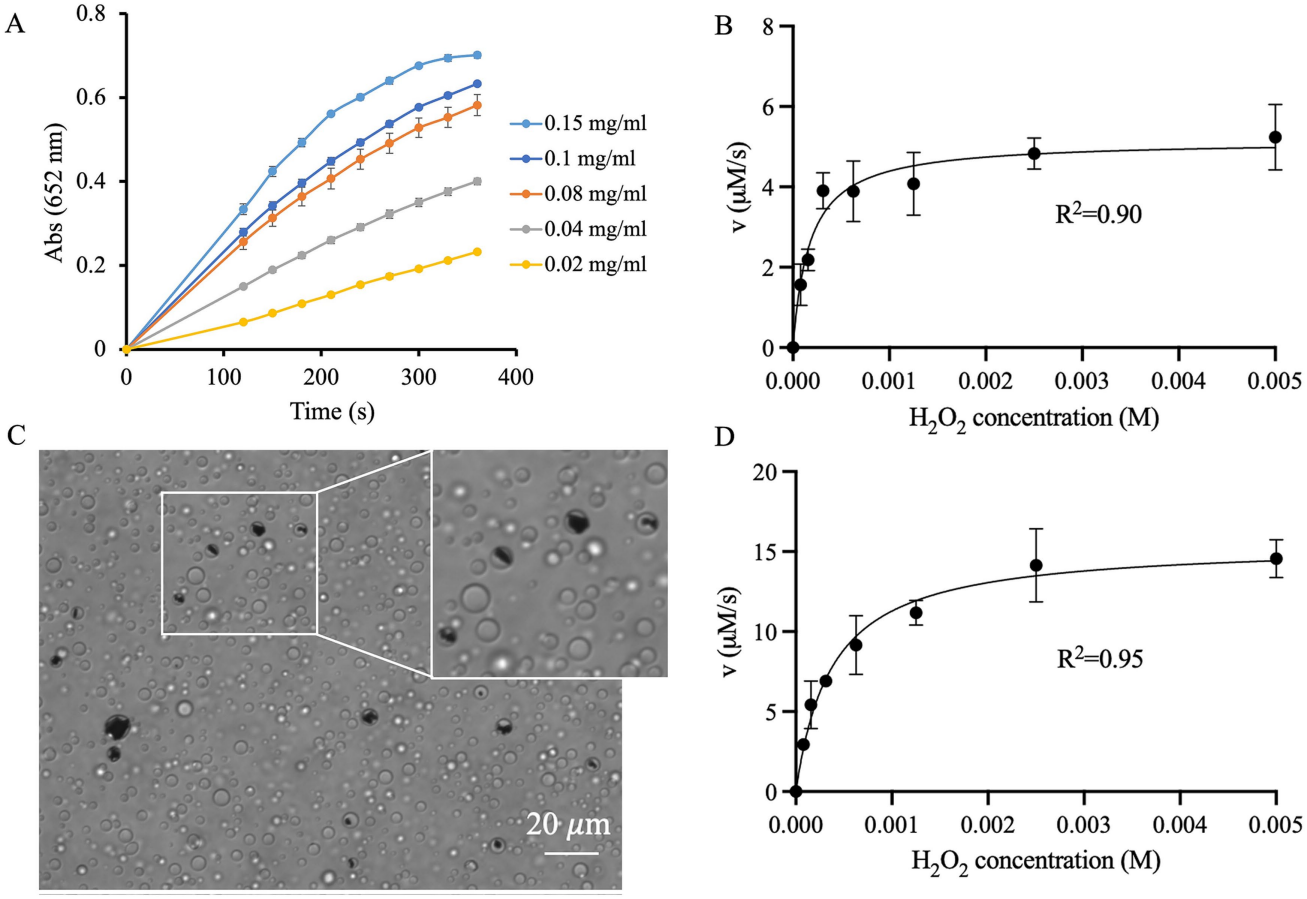
Influence of coacervate droplets on pyrite activity. **(A)** Catalytic activity of pyrite at a concentration range of 0.02–0.15 mg/mL in the presence of 1.0 mg/mL TMB and 0.50 mM H_2_O_2_. **(B)** Catalytic kinetics of 0.15 mg/mL pyrite as a function of H_2_O_2_ concentration. **(C)** Encapsulation of pyrite within droplets. **(D)** Catalytic kinetics of 0.15 mg/mL pyrite after droplets encapsulation.

According to a previous study (Song et al., 2020), the catalytic process of pyrite microparticles can be described in two steps. First, hydroxyl radicals (·OH) are produced from the interaction of pyrite microparticles with H_2_O_2_. Subsequently, these radicals oxidize TMB, resulting in the formation of oxidized TMB (TMBox). It is anticipated that a higher dosage of pyrite microparticles would generate more ·OH, thereby enhancing its catalytic efficiency. This hypothesis is supported by our data (Figure 2A), where the activity is proportional to pyrite concentration. The steady-state kinetic properties were examined by varying the concentration of H_2_O_2_ in a constant concentration of 0.15 mg/mL pyrite-based catalytic system. The oxidation reaction catalyzed by pyrite exhibited a typical Michaelis–Menten curve (Figure 2B). Based on the fitting, the *K*_M_ value for the reaction is 1.73 × 10^−4^ M, and the *V*_max_ is 5.15 ± 0.50 μM/s (Table 1).

**Table 1:** Kinetic parameters for the POD-like activity of pyrite with H_2_O_2_ as substrate.^a^

Samples	*K*_M_ (*M*)	*V*_max_ (μM/s)	*K*_cat_ = *V*_max_/[*E*]^b^ (×10^−3^ s^−1^)	*K*_cat_/*K*_M_ (s^−1^ M^−1^)
Pyrite only	1.73 × 10^−4^	5.15 ± 0.50	4.12 ± 0.40	23.8 ± 2.3
Pyrite in droplet	3.75 ± 10^−4^	15.5 ± 1.4	12.4 ± 1.1	33.1 ± 3.0

a *K*_M_ is the Michaelis constant, *V*_max_ is the maximal reaction velocity, *K*_cat_ is the catalytic constant, and *K*_cat_/*K*_M_ reflects the catalytic efficiency.

b As it is unable to calculate the active site, *E* represents pyrite rather than the active site.

The POD-like activity of pyrite highlights its potential as an early catalyst in the origin of life. In prebiotic chemistry studies, this concept is important because the interaction of pyrite with early Earth environments might have played a role in facilitating primitive metabolic reactions. To explore whether mineral catalytic activity could occur within primitive cells, we investigated the interactions between pyrite and protocells. As expected, after mixing pyrite, ss-oligo and Q-dextran coacervate droplets with TMB (as chromogenic agent) for 5 min, the pyrite microparticles became efficiently encapsulated within the droplets (Figure 2C). However, because the number of droplets exceeded the number of pyrite microparticles, many droplets remained without pyrite.

To evaluate the catalytic activity of encapsulated pyrite, varying concentrations of H_2_O_2_ were added to the samples, and the formation of TMBox was monitored through colorimetric detection. Indeed, the oxidation reaction catalyzed by droplet-encapsulated pyrite also exhibited a typical Michaelis–Menten curve and can be fitted to the Michaelis–Menten equation (Figure 2D). The *K*_M_ value of pyrite microparticles inside droplets is much higher than that of un-encapsulated pyrite (*K*_M_: 3.75 × 10^−4^ M vs. 1.73 × 10^−4^ M, Table 1), indicating a weakened affinity for H_2_O_2_. However, the *V*_max_ of droplet-encapsulated pyrite was approximately 3-fold higher than that of pyrite alone (*V*_max_: 15.5 ± 1.4 μM/s vs. 5.15 ± 0.50 μM/s), resulting in a higher catalytic efficiency (*K*_cat_/*K*_M_: 33.1 ± 3.0 vs. 23.8 ± 2.3).

These results suggest that, although encapsulation within coacervate droplets introduces physical barriers that may reduce the accessibility of H_2_O_2_ to the pyrite surface and restrain the diffusion of ·OH, the local enrichment of TMB within the droplets compensates for these effects and ultimately enhances the overall catalytic efficiency.

### 3.3 The emergence of multiphase droplets catalyzed by pyrite

**Figure 3:**
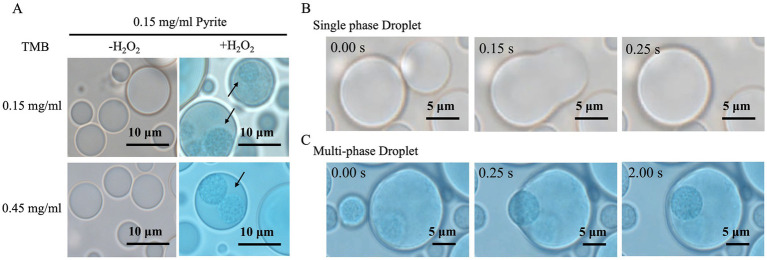
Formation of multiphase droplets. **(A)** Pyrite induces the formation of multiphase droplets in the presence of H_2_O_2_. **(B)** Droplet fusion results in isotropic single-phase droplets in the absence of H_2_O_2_. **(C)** Droplet fusion results in multiphase droplets in the presence of both pyrite and H_2_O_2_. The start time of fusion is recorded as 0 s.

To investigate the impact of mineral catalytic activity on droplets, we observed the color changes and dynamic behavior of droplets under a microscope. Our observations confirmed that, consistent with the catalytic activity assays, the blue TMBox was not observed in the presence of either pyrite or H_2_O_2_ alone (Figure 3A; Supplementary Figure S1), suggesting that the low amount of TMBox formed in these samples (Figure 1B) was insufficient to produce a visible color change. In contrast, in the presence of both pyrite and H_2_O_2_, the droplets turned blue, indicating the formation of TMBox. Notably, the smaller droplets exhibited a darker blue color, suggesting that the reaction proceeded more rapidly in these droplets. Under all experimental conditions, droplet fusions were observed. Interestingly, in the absence of H_2_O_2_, two droplets fused to form a homogeneous droplet (Figure 3B; Supplementary Video S1). However, in the presence of both pyrite and H_2_O_2_, fusion resulted in multiphase droplets, with smaller droplets remaining stably within the larger ones (Figures 3A–C; Supplementary Video S2).

### 3.4 Heterogeneous distribution and mobility of DNA in multiphase droplets

**Figure 4:**
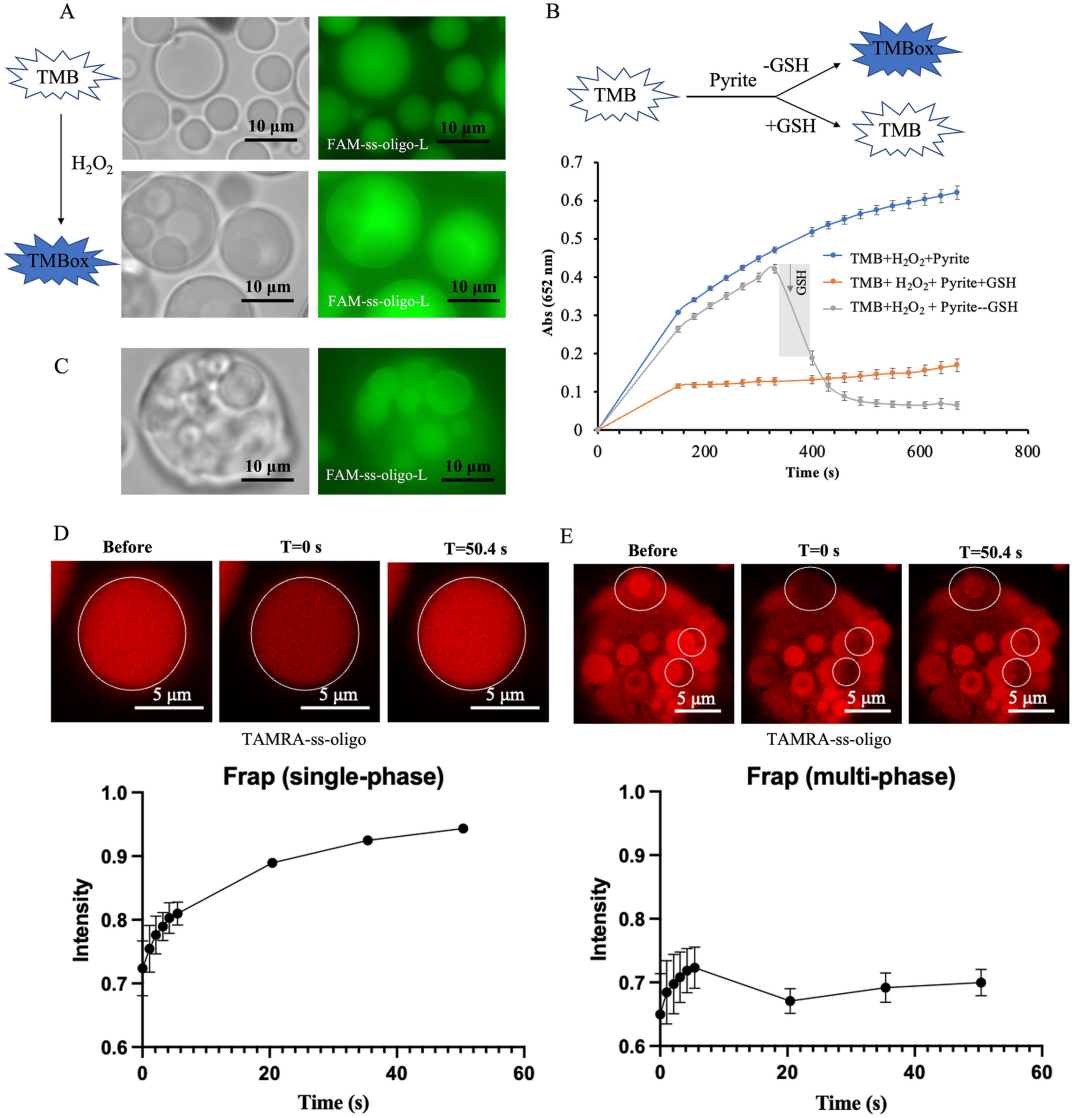
Distribution and mobility of DNA in droplets. **(A)** FAM-ss-oligo-L distribution in single-phase and multiphase droplets. **(B)** Reduction of TMBox by GSH at different times. **(C)** FAM-ss-oligo-L distribution in multiphase droplets after GSH reduction. **(D,E)** (Top) Fluorescence recovery in single-phase and multiphase droplets upon photobleaching. The images show fluorescence intensity before photobleaching (before), immediately after photobleaching (0 s), and after recovery for 50.4 s (50.4 s). (Bottom) Fluorescence recovery of TAMRA-ss-oligo in single-phase and multiphase droplets after photobleaching.

To further reveal the formation mechanism of the multiphase droplets, as well as the distribution of components within the droplets, we initially introduced 6.0 × 10^−3^ mg/mL of FAM-labeled ss-oligo-L (which is excited at 492 nm and emits light at 517 nm) into the droplet system. Ss-oligo-L is about three times longer than that of the ss-oligo used in the droplet system, allowing us to explore how the multiphase structure affects the distribution of exogenous nucleotides. Since TMBox (oxidized TMB) exhibits absorbance across full wavelengths, smaller droplets with higher concentrations of TMBox appeared black under fluorescence microscopy (Figure 4A). To mitigate the color interference of TMBox on FAM-labeled ss-oligo-L, GSH (glutathione) was used to reduce TMBox to TMB (Figure 4B). In the pyrite-H_2_O_2_ system, an increase in absorbance from TMBox production was observed. Introducing GSH at the beginning of the reaction prevented any color development, indicating that GSH inhibited the oxidation of TMB. Also, addition of GSH after 6.0 min of the reaction resulted in a rapid decrease in absorbance, confirming that TMBox was reduced to TMB. Following the reduction of TMBox to TMB by GSH, the multiphase system maintained its stability, and as anticipated, the fluorescence of FAM-labeled ss-oligo-L was clearly observable and was predominantly enriched in the smaller droplets (Figure 4C). The multiphase droplets, as visualized by FAM-labeled ss-oilgo-L, are similar to those by TAMRA-labeled ss-oligo (Figure 3A), indicating that the multiple phase formation is driven by ss-oligo and independent of ss-oligo length.

To investigate the mobility of DNA in single-phase and multiphase droplets, fluorescence recovery after photobleaching (FRAP) experiments were conducted using TAMRA-labeled ss-oligo, which is identical to the ss-oligo used in the droplet system, eliminating any potential differences in mobility due to variation of sequence length. Before photobleaching, an image was captured as a reference for fluorescence intensity, and 0 s was marked after photobleaching (Figures 4D,E). The results showed that after photobleaching, the fluorescence intensity of TAMRA-labeled ss-oligo decreased to 60%–70% of its original value. In single-phase droplets, because of high DNA mobility, TAMRA-labeled ss-oligo quickly recovered fluorescence to >96% after 50.4 s. In contrast, the mobility of TAMRA-labeled ss-oligo in the multiphase droplets was significantly reduced, with fluorescence recovery reaching only 71% after the same amount of time. This suggests that DNA mobility decreases after the formation of the multiphase system, implying that mineral catalysis may promote DNA accumulation and preservation within protocells.

## 4 Discussion

Droplets composed of ss-oligo and Q-dextran formed by liquid-liquid phase separation have been considered a potential protocell model for early life. These coacervate droplets can encapsulate various biomolecules and may have played a role in offering a compartmentalized space for biochemical reactions. Our study demonstrated that these droplets can encapsulate natural pyrite microparticles and increase the catalytic activity of pyrite. Correspondingly, the catalytic activity of pyrite induces a more complex structure in protocells, leading to heterogeneous distribution and mobility of DNA. This may also shed light on the potential mechanisms of storage and stabilization of genetic materials in primary cells.

**Figure 5:**
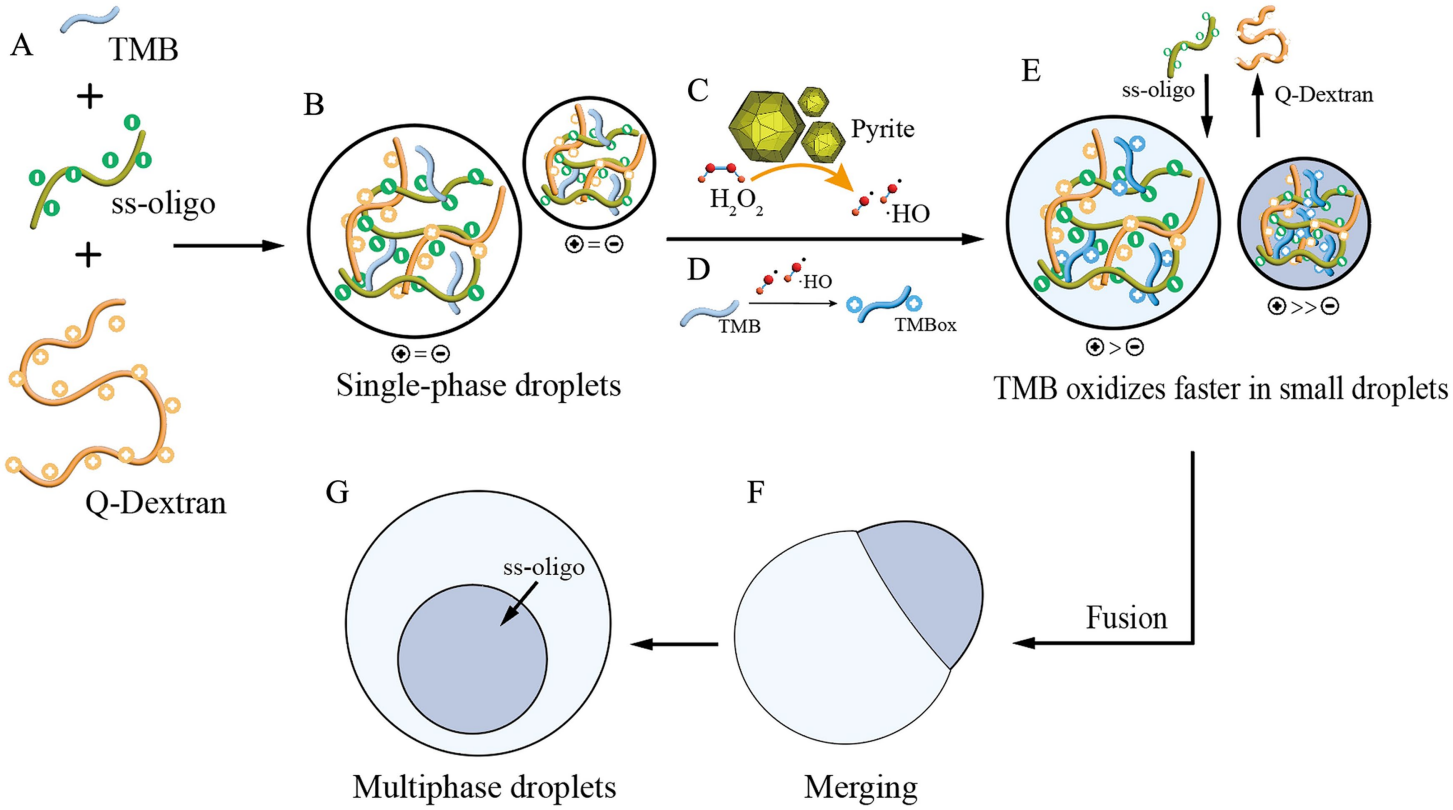
Schematic of pyrite-induced multiphase droplet formation. **(A,B)** TMB, ss-oligo, and Q-dextran assemble to form coacervate droplets with balanced charges. **(C)** Pyrite exhibits peroxidase-like activity, catalyzing the decomposition of H_2_O_2_ to produce hydroxyl radicals (·OH). **(D)** The hydroxyl radicals (·OH) oxidize TMB to its oxidized form, TMB_ox_. **(E)** As TMB becomes oxidized and gains positive charges, the protocells exhibit an overall positive charge. This promotes the capture of negatively charged ss-oligo and the release of positively charged Q-dextran. **(F,G)** Due to varying reaction rates, droplets of different sizes exhibit different densities of TMB_ox_ and ss-oligo. The smaller droplets, with higher positive charge, accumulate more viscous TMB_ox_/ss-oligo, maintaining stability after merging and absorbing DNA from larger droplets.

Although the early Earth was predominantly a reducing environment, mechanical forces on mineral surfaces could have generated reactive oxygen species (ROS), potentially providing the source of H_2_O_2_ (He et al., 2023; Xian et al., 2019). Therefore, we proposed a model for the emergence of these complex protocells (Figure 5). Initially, TMB, ss-oligo, and Q-dextran form a coacervate droplet where the charges are balanced (Figures 5A,B). The peroxidase-like activity of pyrite catalyzes H_2_O_2_ to produce hydroxyl radicals (·OH) (Figure 5C), which then oxidize TMB to its oxidized form, TMBox (Figure 5D). The oxidized TMB gains more positive charges, which generates two major effects: (1) the protocell becomes positively charged (Figure 5E), which is prone to capture more negatively-charged ss-oligo and release more positively charged Q-dextran to maintain a charge balance; (2) the TMBox inside the droplets interact with ss-oligo to form more condensed phase.

These effects result in redistribution of the components inside the droplets as well as the differentiation of the droplets. Due to varying reaction rates, droplets of different sizes form, containing different densities of TMBox and ss-oligo. Smaller droplets, with their higher reaction rates, accumulate more condensed TMBox/ss-oligo phases, which may result in higher viscosity and stability after merging and absorbing DNA from larger droplets (Figures 5F,G). This behavior gives rise to multiphase protocells, and the DNA-enriched subcompartment resembles key features of modern subcellular structures, such as the nucleoid in prokaryotes (Thanbichler et al., 2005). Such compartmentalization may represent a plausible prebiotic mechanism for molecular sorting, stabilization, and selective compartmentalization.

## 5 Conclusion

This study demonstrates a dual impact of interaction between protocells and pyrite: protocells enhance the catalytic efficiency of pyrite, while pyrite catalysis promotes the emergence of life-like structural features in the protocells. These findings suggest a synergistic relationship between primitive cellular systems and mineral catalysts, offering insights into the evolution of early life.

The enzyme-like catalytic activities of minerals have been increasingly reported, suggesting their potential roles as fundamental catalysts in early primitive biochemical processes. It is estimated that approximately 420 different types of minerals existed on prebiotic Earth (Hazen, 2013). The surfaces of these minerals played crucial roles in protecting, selecting, concentrating, templating, and catalyzing pre-life organic molecules (Dong et al., 2022; Hazen et al., 2008), facilitating the chemical reactions necessary for the emergence of life. Future studies should explore the interactions between protocells and other minerals present on prebiotic Earth to better understand their contributions to the origin and evolution of early life.

## Data Availability

The original contributions presented in the study are included in the article/Supplementary material, further inquiries can be directed to the corresponding authors.
