# Supplementary figures and images for: Pyrite as a catalyst for the emergence of multiphase primitive cells
Source: Front Microbiol. 2026 Jan 8;16:1747422. doi: 10.3389/fmicb.2025.1747422 (PMC12823477; doi:10.3389/fmicb.2025.1747422)

Supporting Information：

**
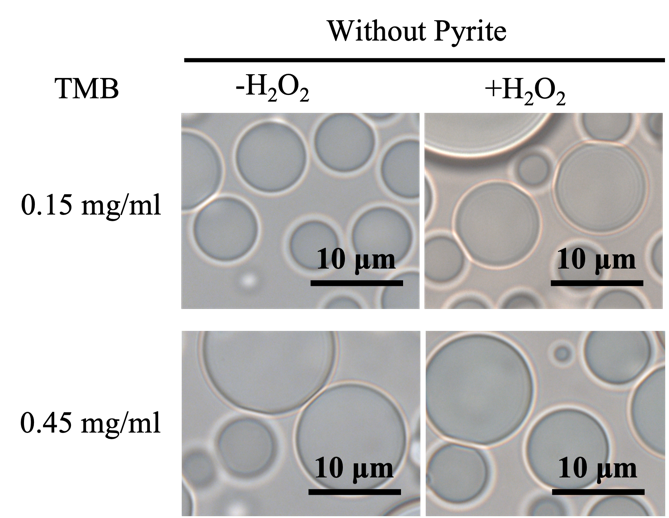
**

**Figure S1. Morphological changes of droplets without pyrite**

Supplement: Supplementary file 1 [file Supplementary_file_1.docx]
